# Exploring Lignans, a Class of Health Promoting Compounds, in a Variety of Edible Oils from Brazil

**DOI:** 10.3390/foods11101386

**Published:** 2022-05-11

**Authors:** Roberta Tardugno, Nicola Cicero, Rosaria Costa, Vincenzo Nava, Rossella Vadalà

**Affiliations:** 1 Science4Life s.r.l., Spin Off Company, University of Messina, 98122 Messina, Italy; roberta.tardugno@gmail.com (R.T.); ncicero@unime.it (N.C.); 2 Dipartimento di Scienze Biomediche, Odontoiatriche e delle Immagini Morfologiche e Funzionali (Biomorf), University of Messina, 98122 Messina, Italy; vincenzo.nava@unime.it (V.N.); rossella.vadala@unime.it (R.V.); 3 Consorzio di Ricerca sul Rischio Biologico in Agricoltura (Co.Ri.Bi.A.), 90100 Palermo, Italy

**Keywords:** lignans, Brazilian oils, vegetable oils, olive oil, oil flavor, volatiles, SPME-GC, LC-MS

## Abstract

Lignans, a group of polyphenols, have been identified in eight cold pressed oils from fruits, nuts, and seeds, retrieved from the Brazilian market. The oils under investigation were avocado, Brazilian nut, canola, coconut, grapeseed, macadamia, palm, and pequi. Olive oil was selected as a reference oil, since numerous data on its lignan content are available in literature. The qualitative and quantitative profiles were obtained, after extraction, by means of UFLC-ESI-MS/MS analyses. The total lignan content showed a high variability, ranging from 0.69 mg·Kg^−1^ (pequi) to 7.12 mg·Kg^−1^ (grapeseed), with the highest content registered for olive oil. Seven lignans were quantified, matairesinol and pinoresinol being the most abundant. The LC-MS/MS method was validated, showing linearity in the range of 12.5–212.5 mg·Kg^−1^, LOD in the range of 0.18–11.37 mg·Kg^−1^, and LOQ in the range of 0.53–34.45 mg·Kg^−1^. Additionally, part of the study was focused on the evaluation of the flavor profile, this being a key element in consumers’ evaluations, by means of HS-SPME-GC. In total, 150 volatile compounds were determined in the eight oils, with identified fractions ranging from 91.85% (avocado) to 96.31% (canola), with an average value of 94.1%. Groups of components contributed characteristically to the flavour of each oil.

## 1. Introduction

Numerous are the cold-pressed oils used in Brazil for food consumption, and more recently for cosmetic and pharmaceutical formulations in developed countries. The latter use is widely justified by their content in bioactives, mostly health promoting compounds, in particular when the oils are extracted from nut tree species [1]. In the wide scenario of tropical fruits harvested and consumed as such in Brazil, some are exploited for the obtainment of the oil: Brazil nut, macadamia, and pequi (oils extracted from seeds); and avocado and coconut (oils extracted from the pulps). In addition, although not native to the Brazilian country, palm, canola, and grapeseed oils have a great commercial importance as well. The typical research focus of oil investigation is the elucidation of their lipidic profile, namely through the determination of triacylglycerols, fatty acids, phospholipids, sterols [2,3]. This also seems to be the case with oils from the present study [4,5,6,7,8,9,10,11]. As evidenced by Cicero et al. (2018), embedded within the fatty matrix there are indeed numerous minor components that often play an important role in good health maintenance [12]. Among these, polyphenols, squalene, and micronutrients have been already determined in the present oils [12]. As a continuation of this previous study, the same commercial oils have been subjected to chemical investigation to assess the content of lignans, which are a group of secondary metabolites belonging to the class of polyphenols. Lignans are produced by plants of various genera and species and are formed by the union of two cinnamic acid residues or their equivalents. They are found in conifers, but also in plants used as food, although their concentrations are lower. The highest concentrations were found in flaxseed and sesame, while the lowest were in vegetables of the Brassicaceae family, nuts, and cereals [13]. The lignans secoisolariciresinol and matairesinol were also determined in some berry species and strawberries [14,15]. Most of the interest in lignans is due to their potential application in the pharmaceutical and nutraceutical fields. They exhibit a diverse range of beneficial effects, such as antioxidant, antiviral, antidiabetic, anti-obesity, and protection against cardiovascular disease [14]. Currently, there are no recommendations for a daily intake of lignans for disease prevention, but several studies suggest that a dietary intake of lignans may improve degenerative diseases [14]. Lignans are the most abundant phenolic compounds in virgin olive oils after secoiridoids. Their concentration depends mainly on the variety, while the milling process does not significantly affect their amount. The presence of the latter compounds allowed EFSA to approve the health claim “Olive oil polyphenols contribute to the protection of blood lipids against oxidative stress” [16,17]. In this light, due to the potential pharmacological properties of lignans, and given the diversity of plant matrices where they are present, it seemed appropriate to deepen the knowledge on the distribution in nature of this class of compounds.

To this aim, lignan polyphenols were investigated in a variety of cold-pressed gourmet oils obtained from nuts, berry fruits, and seeds available in the Brazilian market. Extra virgin cold pressed olive oil has been analysed as a benchmark. Finally, to complete the phytochemical survey, part of the study was focused on the evaluation of the flavor profile, this being a key element in consumers’ evaluation, which can also provide insights about oils shelf-life.

## 2. Materials and Methods

### 2.1. Chemicals and Solvents

Gallic acid, hydroxymatairesinol, isolariciresinol, lariciresinol, matairesinol, pinoresinol, secoisolariciresinol, and secoisolariciresinol glucoside were provided by Sigma-Aldrich (Milan, Italy). Formic acid (HCOOH) and HPLC-MS solvents such as methanol (MeOH) and water (H_2_O) were from Sigma-Aldrich (Milan, Italy).

### 2.2. Oil Samples

Samples of commercially available vegetable oils from pulps and seeds were purchased from different local markets in Belo Horizonte (Minas Gerais, Brazil). Specifically, six oils were extracted from seeds: coconut (*Cocos nucifera* L.), Brazil nut (*Bertholletia excelsa* Humb. and Bonp), macadamia (*Macadamia integrifolia* Maiden and Betche), pequi (*Caryocar brasiliense* Cambess.), canola (*Brassica napus* L.), and grapeseed (*Vitis vinifera* L.); other two oils were extracted from fruit pulps: avocado (*Persea americana* Mill.) and palm (*Elaeis guineensis* Jacq.). For a detailed description of samples, the reader has to refer to [12]. Each sample was stored in a cool and dark place until the time of analysis.

### 2.3. Extraction of Lignans

10 g of oil were extracted in 10 mL of methanol/water 80:20 solution. The mixture was vortexed at 5000 g for 1 min and then centrifuged at 5000 rpm for 5 min. The supernatant was separated, and the extraction was repeated two more times with fresh MeOH/H_2_O 80:20 solution. The alcoholic solution was evaporated under reduced pressure at approximately 35 °C and the residue was redissolved with 1 mL of MeOH/H_2_O 60:40 solution [12,18]. The diluted extract was then filtered into an HPLC vial using a 0.45 µm cellulose acetate syringe filter (Whatman, Maidstone, UK) and stored in the dark at +4 °C before injection into the HPLC system.

### 2.4. HPLC-MS/MS-Analysis

The analyses were performed with a UFLC (Ultra-Fast Liquid Chromatography) coupled to LCMS-8040 detector (Shimadzu, Kyoto, Japan) with Agilent Poroshell C18 (100 × 2.1 mm I.D., 1.8 µm). The temperature of the column compartment was set at 40 °C and the injection volume was set at 2.0 µL. The compounds were eluted in gradient mode using a mobile phase consisting of water containing 0.1% formic acid (*v*/*v*) (mobile phase A) and methanol containing 0.1 formic acid (*v*/*v*) (mobile phase B). Separation was achieved by gradient elution as follows: 0–6.5 min from 35 to 50% B, 6.5–7.5 min from 50 to 53% B, 7.5–10 min from 53 to 98%, 10–13 min 98% B hold, 13–16 from 98 to 35% B. Subsequently, a follow-up time of 5 min at 35% B was applied. The LCMS-8040 triple quadrupole analyzer with ESI source was used with the following operating conditions: nebulizing gas (nitrogen) 3.0 L/min; drying gas (nitrogen) 15 L/min; heating block temperature 400 °C; desolvation line temperature 250 °C; CID gas 230 kPa, interface voltage 3.5 kV; detector voltage 1.80 kV. Collision energy (CE) and fragmentor voltage were optimized for each compound class by injecting standard solutions in MeOH directly into the mass spectrometer. Detection of analytes was performed in both positive and negative ion modes by Multiple Reaction Monitoring (MRM). All measurements were performed in triplicate. Compounds were identified using the calculated exact mass and retention time of each target compound, which are listed in Table 1. For quantitative purposes, an external calibration procedure was performed. Specifically, standard stock solutions were prepared individually in MeOH/H_2_O (80:20, *v*/*v*) at a concentration of approximately 1000 mg/L. A single working solution with a concentration of 125 mg/L was obtained by mixing known amounts of each standard solution in a 100 mL volumetric flask and diluting with MeOH to the mark. Then, several standard solutions (from 0.25 to 25 mg/L) were prepared by serial dilution and injected in six replicates to generate six calibration curves of selected lignans; gallic acid was used as an internal reference standard (from 0.1 to 10 mg/L). Peak areas were calculated and plotted against the corresponding concentrations of the standard compounds using linear least squares regression to generate standard curves [12,19,20,21].

### 2.5. Flavor Profile

The flavor fingerprint was determined by headspace solid-phase microextraction (HS-SPME) followed by gas chromatography (GC) coupled to FID and MS detection systems. The SPME apparatus consisted of a DVB/Carbon WR/PDMS 80 μm fiber coating (Agilent Technologies, Santa Clara, CA, USA). Following method optimization, 5 mL of oil were put into a 10 mL headspace crimped vial, added with 0.5 g of table salt, and stirred. After a presaturation period (20 min at 50 °C), the fiber was exposed for 30 min at 50 °C; stirring speed: 300 rpm; desorption: 5 min in GC injector (250 °C). GC-FID analyses were performed on a GC-2010 (Shimadzu, Milan, Italy), equipped with a Zebron-5 ms capillary column, 30 m × 0.25 mm ID × 0.25 μm d_f_ (Phenomenex, Torrance, CA, USA). The oven program temperature was from 50 °C (1 min) to 250 °C (held 1 min) at 4 °C min^−1^, to 300 °C (held 10 min) at 10 °C min^−1^. The injection port was equipped with a narrow inlet liner (0.75 mm ID, Agilent Technologies). Sample injection took place in splitless mode, with 5 min sampling time, then split ratio of 20:1. Carrier gas (He, 210.0 KPa, pressure control mode) was used at a linear velocity of 30 cm s^−1^. FID detector (300 °C); gas flows were 40 mL min^−1^ for hydrogen and 400 mL min^−1^ for air. Data handling was performed by means of *GCsolution 2.32* software.

For mass spectrometric analyses, a GCMS-TQ8030 (Shimadzu) was used. The instrument was equipped with the same Zebron-5 ms capillary column and operated at the same experimental conditions reported above. The MS set-up was as follows: ion source, 200 °C; interface temperature, 250 °C; electron multiplier voltage, 1.0 kV; mass range, 40–400 amu. For qualitative analysis, mass spectral databases were: FFNSC2 (Wiley), Adams 4th edition (Allured), and NIST11, each provided with Retention Index parameters, as an aid to identification. Experimental Retention Indices were measured by injecting an HS-SPME extract from a laboratory-made solution of n-paraffins ranging from n-hexane to n-hexadecane (concentration range: 5.0–50.0 ppm). Specifically, to avoid the SPME fiber oversaturation caused by lower boiling point paraffins, the solution was prepared by adding to a 25 mL volumetric flask, 0.125 mg/each of C6, C7, C8, and C9; 0.25 mg/each of C10, C11, and C12; 12.5 mg/each of C13, C14, and C15; and finally adding C16 as main solvent until reaching volume.

## 3. Results

### 3.1. Lignans

Lignans are molecules with a wide range of utilities in food/nutraceutical fields. Indeed, lignans exert a number of bioactivities on human health including antioxidant, antimicrobial, anti-inflammatory, and many others. Scientific data on lignans are reported in more than 100 peer-reviewed articles [22]. Several possible mechanistic explanations for the observed bioactivities such as anti-oxidation and gene suppression have been reported [23]. In addition to their biological functions, lignans play a fundamental role in the establishment of an organoleptic profile. Their presence contributes to the unique taste of food and spirits, in this case, of edible oils, due to their bitterness [24,25]. From an analytical point of view, the best separation of lignans was here obtained with the mobile phase MeOH/H_2_O (both containing 0.1% formic acid) on a Poroshell C18 column thermostatted at 40 °C under gradient elution at a flow rate of 0.3 mL/min. Each compound was identified by a qualification transition and by one or (if available) two confirmatory MS–MS transitions. The HPLC-QqQ fragments found were compared with literature data [19,26,27,28]. Linearity over the range of concentrations tested was optimal, exhibiting r2 > 0.9997 for all reference standards, as shown in Table 2.

The limit of detection (LOD) and the limit of quantification (LOQ) were experimentally determined by injecting serial dilutions of a standard solution to reach a signal-to-noise (S/N) ratio of 3 and 10, respectively. LOD values ranged from 0.21 to 11.37 μg/mL, while the LOQ value ranged from 0.81 to 34.11 μg/mL (Table 2), indicating a good sensitivity of the method. The developed method was first applied to the quali-quantitative determination of lignans in cold pressed extra virgin oil, then seed and fruit pulp oils from the Brazilian market. Quantitative data for the detected lignans are presented in Table 3, expressed as mg/kg.

As expected, the concentrations showed a remarkable variability among the samples studied. The total amount of lignan constituents ranged from 0.69 to 10.39 mg/kg, in the following decreasing order: olive oil (10.39 mg/kg) > grape seed oil (7.12 mg/kg) > macadamia oil (4.50 mg/kg) > canola oil (4.18 mg/kg) > avocado oil (3.87 mg/kg) > palm oil (1.00 mg/kg) > Brazil nut oil (0.94 mg/kg) > coconut oil (0.89 mg/kg) > pequi oil (0.69 mg/kg). Pinoresinol (9.11 mg/kg) and lariciresinol (1.28 mg/kg) were found as lignan components of EVOO and their concentrations were within the ranges reported in the literature [12,14]. Grapeseed oil was the second richest oil in lignans (total 7.12 mg/kg), with pinoresinol as the predominant analyte in accordance with the literature [29,30]. Moreover, the presence of all seven target lignans were detected for the first time in this oil. Macadamia, canola, avocado, palm, Brazilian nut, coconut, and pequi cold-pressed gourmet oils showed the presence of phenolic lignan components. The total lignan content found in macadamia oil was 4.50 mg/kg with pinoresinol (1.92 mg/kg) and lariciresinol (0.91 mg/kg) as the most abundant lignans. In general, the polyphenolic profile of macadamia oil shows an important variability due to several factors (growing conditions, variety, and location) ranging from about 2 to 120 mg/kg [10,12]. In canola, the total lignan content was 4.18 mg/kg with lariciresinol (1.51 mg/kg) and pinoresinol (1.36 mg/kg) as the main lignans. Compared to other seed oils, canola presents higher amounts of polyphenols up to about 110 mg/kg [10,12]. In avocado, total lignans were lower than 3.87 mg/kg, with matairesinol (0.87 mg/kg) and pinoresinol (0.77 mg/kg) as the prevailing compounds. Lignans in avocado fruits were previously reported by Rodríguez-García and coworkers (2019) [14]. Palm oil reported only trace amounts of lignans, namely 0.70 mg/kg pinoresinol and other constituents at levels lower than LOQ. Similarly, to palm oil, in Brazil nut the total lignan content was 0.94 mg/kg (about 0.1–0.2 mg/kg of matairesinol and secoisolariciresicol), confirmed elsewhere [14]. The coconut oil lignan content resulted in being 0.89 mg/kg, with about 0.1–0.25 mg/kg of isolariciresinol, lariciresinol, matairesinol, and pinoresinol. Only negligible amounts of lignans, namely isolariciresinol, lariciresinol, matairesinol, and pinoresinol, were detected in pequi oil (0.69 mg/kg). Studies on the phenolic compounds profile of pequi are scarce in the literature, the total phenolic contents among different pequi species reported variability among the samples [31].

### 3.2. Flavor

Table 4 reports the volatile compounds (VOCs) that were determined in at least two oil samples. However, around 150 different components were determined in total in the eight gourmet oils. According to a previously validated procedure, volatiles were quantified as mg/kg [32,33]. Expanded uncertainty, standard error, and asymmetry have been also measured and reported in Table S1.

**Figure 1:**
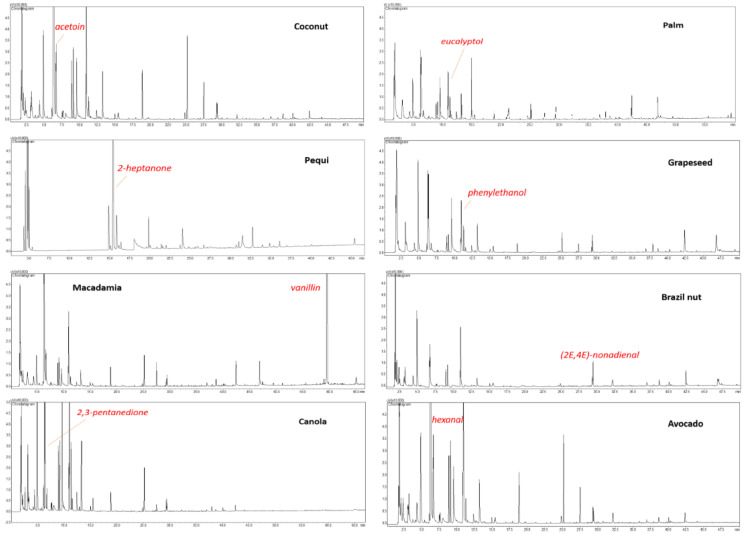
HS-SPME-GC-FID chromatograms of the eight Brazilian cold-pressed oils.

Flavor chromatographic fingerprints are shown in Figure 1, which allows us to perceive the complexity of the volatile matrices through the crowded chromatographic space. In addition, by comparing the single chromatograms, it can be roughly understood which oils have a richer aroma. In particular, the highest content of VOCs was found in canola, coconut, and avocado oils, whereas the poorest fingerprints were obtained for macadamia, pequi, and Brazil nut. The total identified fraction ranged from 91.85% (avocado) to 96.31% (canola), with an average value of 94.1%. Each oil showed a characteristic label that in general appears to be partially superimposable to that found in the scarce number of previous works on target oil flavours [34,35,36,37]. Terpenes, including limonene, (E)-caryophyllene, farnesol, β-bisabolene among others, were detected in avocado; trace amounts of fragrant lactones, such as δ-nonalactone, δ-decalactone, and δ-dodecalactone were determined in coconut. Phenyl compounds and acetates, i.e., phenethyl alcohol, were detected in grapeseed oil. Some furan derivatives along with oxygenated monoterpenes were isolated from palm oil. Impact odorants, e.g., vanillin and cinnamaldehyde, along with some sesquiterpenes, were determined in macadamia. Furthermore, free fatty acids (linear and branched, C3–C12) were distributed randomly in the different oils. Finally, traces of sulphides were found in pequi. The flavour of canola showed to be the most comprehensive, reporting most of the volatiles afore discussed. Conversely, Brazil nut did not show any particular character.

## 4. Discussion

### 4.1. Lignans

#### 4.1.1. Method Optimization

The main aspect of this work is the development of a single analytical method for the extraction, analysis, and comparison of different vegetable oils with respect to their lignan content. The extraction parameters, including solvent composition and extraction procedure, were optimized to obtain the most proficient method. For extraction optimization, different solvents (methanol, ethanol, isopropanol), solvent/water ratios, and methodologies (static and dynamic maceration, ultrasonication), were tested. Among these conditions, methanol/water 80:20 centrifuged solution, freshly prepared in triplicate, proved to be the most efficient method in terms of selectivity, time, and amount of solvents [12,18]. The chromatographic parameters, including column choice, mobile phase composition, flow rate, and temperature, were optimized to obtain the best results in terms of resolution, efficiency, and analysis time. With respect to column selection, the Poroshell C18 was superior than a conventional C18 in terms of peak resolution, reduction of the system backpressure, and analysis duration [38].

The mobile phases (methanol–formic acid solution and acetonitrile–formic acid solution) were optimized. The presence of formic acid in both the mobile phases was also evaluated, resulting in an improvement of the peak shapes and of the ESI-MS intensity signal. In consideration of the chromatographic run, flow rate adjustments resulted essential. Flow rates from 0.2 to 0.8 mL/min were tested, with a lower resolution observed for higher flow rates (i.e., 0.8 mL/min). The last parameter optimized was the temperature of analysis, tested in the range from room temperature (uncontrolled) up to 40 °C: a reduction of mobile phase viscosity and of column pressure was measured, consequently improving the separation of the lignan constituents [20,39]. Considering the complexity and variability of the matrices selected for this study, the chromatographic performance can be considered satisfactory.

#### 4.1.2. Occurrence and Bioactivity

According to literature data, extra virgin olive oil (EVOO) presents a considerable amount of lignans, therefore justifying its choice as a model oil for comparison [40]. The results obtained here support this finding, since the highest concentration of lignans has been precisely determined in the EVOO sample. These data confirm the importance of the EVOO polyphenolic profile for the Mediterranean diet and related health benefits. Moreover, the lignans concentration was within the range reported in the literature [19,29]. Grapeseed oil has been previously reported as a rich source of phenolics, displaying a powerful antioxidant effect that corroborates the nutraceutical value of this winemaking industry by-product [41]. The literature demonstrates that macadamia, canola, avocado, palm, Brazilian nut, and pequi oils are sources of antioxidant polyphenols. Nonetheless, as is known to most, phenolics concentration is dramatically dependent on a variety of factors, such as pedoclimatic conditions, cultivar type, processing, and storage [10,12,14,31,42,43,44]. Lignans are dimers derived from hydroxycinnamylic alcohols. They are the main building blocks of lignin. Pinoresinol is the parent molecule of a variety of lignans such as secoisolariciresinol, matairesinol, and podophyllin. The latter is a characteristic constituent of *Podophyllum* spp., which can reach up to 60% of the lignan fraction present in their resin. Podophyllin exerts a potent cytotoxic activity, being its semisynthetic derivative, namely etoposide, a well known and effective anticancer drug. The antineoplastic action of lignans has to be attributed to their fused lactones ring, which is capable of inhibiting topoisomerases, the enzymes responsible for DNA replication.

### 4.2. Flavor

The HS-SPME-GC analyses evidenced a quite rich flavor in all the oils investigated. As can be seen from Table 3, the volatiles can be grouped in specific main classes: aliphatic aldehydes, ketones, acids, and alcohols. Worth mentioning are: Hexanal, found in five different oils (1.91–19.28 mg/kg) and has been reported as an oxidative marker in soybean, corn, and olive oils [45]. The literature supports headspace-SPME as the elective technique for the determination of hexanal, a molecule prone to further oxidation. 2,3-Pentanedione, determined in canola and macadamia oils, is a naturally occurring odorant, often added as a synthetic flavouring agent to canola oil and popcorn (buttery flavor) [46]. Benzaldehyde, common to four oils, has been previously reported as an aroma-active constituent of some seed oils [47]. In general, alkenals such as (2E,4E)-nonadienal, (2E,4E)-decadienal, (2E)-decenal, (2E)-hexenal, (2E)-heptenal, and the above mentioned hexanal are regarded as markers of oxidation [48]. In particular, nonanal, (2E)-decenal, and heptanal are oxidation products of oleic acid; hexanal, (2E)-nonenal, and (2E,4E)-decadienal are formed by linoleic acid oxidation; benzaldehyde and (2E,4E)-heptadienal are oxidation products of linolenic acid [48]. Nonetheless, only in some cases the content of these volatiles was so high to suggest a potential rancidity involvement. For instance, when taking into account the contents of hexanal, (2E)-decenal, and (2E,4E)-decadienal, avocado oil appeared as the most oxidized oil, followed by Brazil nut (see hexanal, benzaldehyde, and (2E,4E)-nonadienal amounts). Canola oil reported only a noticeable concentration of (2E,4E)-decadienal. Besides discussing the VOCs correlated to oxidation phenomena, it is useful to specify that the manufacturer declares that the oils were extracted by cold pressure, thus ensuring the preservation of their nutritional value. The presence of VOCs regarded as oxidation markers, although limited to low concentrations with few exceptions, has to be attributed to all those processes of natural oxidation occurring during storage, transport, and laboratory handling, when accidental exposure to air and light might trigger such reactions [49]. Moreover, such findings corroborate the authenticity of the oils themselves, confirming the absence of preliminary heat treatment and the addition of preservatives.

## 5. Conclusions

Although most of the plants reported here have been previously investigated about their composition, many of their bioactive constituents remain to be fully identified and characterized. In some cases, the number of reports appears limited, as seen for lignans. Therefore, the aim of this work was to fill the gap in our knowledge on phytochemicals present in the oils extracted from plants native to Brazil through the application of a UFLC-MS/MS method. The results obtained in this study indicated the presence of lignans in eight cold-pressed edible oils. In most cases, lignan constituents were identified and quantified for the first time, showing an intrinsic variability of the total lignan content. The highest contents were found in olive oil and grapeseed oil, followed by macadamia, canola, and avocado. The analytical method proved to be a reliable tool for the determination of lignan content in edible oils and could therefore be validated and used for the selection of edible oils rich in antioxidant and anticarcinogenic lignan phenolics, as well as for nutritional intake recommendations and corresponding labels. Additionally, as a parameter affecting consumers’ appreciation, the aromatic fraction of each oil was investigated by means of HS-SPME-GC, leading to the determination of about 150 volatiles. Although some of them are regarded as oxidation markers, their levels were well below the limits of rancidity. Interestingly, each oil showed a characteristic pattern of volatiles/odorants, widely correlated to cold pressure extraction.

## Figures and Tables

**Table 1:** Lignan reference standards selected for the investigation in gourmet edible oils by HPLC-MS/MS (positive and negative mode).

Compound Name	t_R_	Precursor Ion (*m/z*) ^a^	Product Ions (*m/z*)
Hydroxy matairesinol	2.82	373	355, 217, 173
Isolariciresinol	11.04	359	344, 313, 91
Lariciresinol	4.31	359	329, 313, 91
Matairesinol	6.46	357	342, 137, 83
Pinoresinol	5.96	341 (+)	323, 271, 137
Secoisolariciresinol	4.63	361	165, 121
Secoisolariciresinol diglucoside	2.4	685	523, 361

^a^ (+) = positive mode.

**Table 2:** Linearity and sensitivity data obtained from UPLC-MS/MS analysis of lignans reference standards ^a^.

Compound	Linearity Range (μg/mL)	Slope (a)	Intercept (b)	r^2^	LOD (μg/mL)	LOQ (μg/mL)
Hydroxy matairesinol	21.2–212.5	53.27	−10.02	0.997	0.93	2.83
Isolariciresinol	12.5–125	134.5	−1.4	0.998	6.25	18.94
Lariciresinol	15–150	882.1	3.91	0.999	0.18	0.53
Matairesinol	12.5–125	171.3	13.8	0.999	11.37	34.45
Pinoresinol	14–137.5	681.9	−10.03	0.999	0.63	1.9
Secoisolariciresinol	15–150	11.45	1.02	0.998	0.21	0.62
Secoisolariciresinol diglucoside	21.2–212.5	61.1	6.18	0.999	0.32	1.64

^a^ For each calibration curve the equation is y = ax + b, where y is the peak area, x the concentration of the analyte (μg/mL), a is the slope, b is the intercept and r^2^ the correlation coefficient.

**Table 3:** Concentration levels of lignans in edible gourmet oils by HPLC-MS/MS, expressed as mg/kg.

Compound	Olive (mg/kg)	Avocado (mg/kg)	Brazil nut (mg/kg)	Canola (mg/kg)	Coconut (mg/kg)	Grapeseed (mg/kg)	Macadamia (mg/kg)	Palm (mg/kg)	Pequi (mg/kg)
Hydroxy matairesinol	n.d.	0.44 ± 0.01	n.d.	0.13 ± 0.0	<LoD	0.93 ± 0.02	0.24 ± 0.0	n.d.	<LoD
Isolariciresinol	n.d.	0.63 ± 0.03	0.20 ± 0.0	0.34 ± 0.02	0.26 ± 0.01	0.99 ± 0.06	0.43 ± 0.03	0.20 ± 0.0	0.25 ± 0.01
Lariciresinol	1.28 ± 0.14	0.58 ± 0.03	0.20 ± 0.0	1.51 ± 0.18	0.22 ± 0.01	1.07 ± 0.09	0.91 ± 0.08	0.30 ± 0.01	0.18 ± 0.01
Matairesinol	n.d.	0.87 ± 0.04	0.21 ± 0.0	0.50 ± 0.01	0.16 ± 0.0	1.07 ± 0.13	0.51 ± 0.01	<LoD	0.08 ± 0.0
Pinoresinol	9.11 ± 0.45	0.77 ± 0.06	0.12 ± 0.0	1.36 ± 0.12	0.24 ± 0.0	1.68 ± 0.12	1.92 ± 0.21	0.70 ± 0.03	0.18 ± 0.01
Secoisolariciresinol	n.d.	0.24 ± 0.0	0.21 ± 0.0	0.21 ± 0.01	<LoD	0.58 ± 0.03	0.33 ± 0.03	<LoD	n.d.
Secoisolariciresinol diglucoside	n.d.	0.34 ± 0.04	n.d.	0.13 ± 0.0	0.01 ± 0.0	0.80 ± 0.06	0.16 ± 0.0	n.d.	<LoD
Total	10.39	3.87	0.94	4.18	0.89	7.12	4.50	1.00	0.69

n.d. = not detected.

**Table 4:** Key volatiles sampled by HS-SPME in oil samples. Values are expressed as mg/Kg ± SD (*n* = 3).

nr.	Compound	RI ^¤^	RI ^†^	Canola $\overset{-}{x}$ ± SD (mg/Kg)	Avocado $\overset{-}{x}$ ± SD (mg/Kg)	Coconut $\overset{-}{x}$ ± SD (mg/Kg)	Palm $\overset{-}{x}$ ± SD (mg/Kg)	Grapeseed $\overset{-}{x}$ ± SD (mg/Kg)	Macadamia $\overset{-}{x}$ ± SD (mg/Kg)	Brazil Nut $\overset{-}{x}$ ± SD (mg/Kg)	Pequi $\overset{-}{x}$ ± SD (mg/Kg)
1	Butanal	610	607	2.02 ± 0.01	2.76 ± 0.04	n.d.	5.53 ± 0.04	n.d.	n.d.	n.d.	n.d.
2	Butanol	620	617	n.d.	n.d.	1.23 ± 0.02	n.d.	n.d.	n.d.	5.21 ± 0.04	n.d.
3	2-Methylbutanal	663	662	3.42 ± 0.03	n.d.	n.d.	4.38 ± 0.02	n.d.	n.d.	n.d.	n.d.
4	2,3-Pentanedione	699	695	6.12 ± 0.02	n.d.	n.d.	n.d.	n.d.	12.36 ± 0.03	n.d.	n.d.
5	Pentanal	701	696	1.64 ± 0.01	1.80 ± 0.03	n.d.	2.33 ± 0.04	n.d.	n.d.	n.d.	n.d.
6	Acetoin	721	716	n.d.	3.65 ± 0.06	7.78 ± 0.14	n.d.	n.d.	n.d.	n.d.	5.84 ± 0.06
7	Isopentyl alcohol	728	729	n.d.	n.d.	0.72 ± 0.01	n.d.	1.79 ± 0.02	n.d.	n.d.	n.d.
8	Pentanol	754	759	n.d.	n.d.	1.12 ± 0.03	n.d.	n.d.	n.d.	1.66 ± 0.02	n.d.
9	2,3-Butanediol	792	788	n.d.	2.02 ± 0.03	5.55 ± 0.20	n.d.	n.d.	n.d.	n.d.	n.d.
10	Hexanal	801	801	1.91 ± 0.01	19.28 ± 0.32	3.33 ± 0.04	9.35 ± 0.06	n.d.	n.d.	12.31 ± 0.12	n.d.
11	2-Hexanol	804	802	n.d.	0.87 ± 0.01	n.d.	n.d.	n.d.	n.d.	16.53 ± 0.16	n.d.
12	(2E)-Hexenal	853	850	0.89 ± 0.00	n.d.	n.d.	5.63 ± 0.04	n.d.	n.d.	n.d.	n.d.
13	2-Heptanone	902	898	n.d.	n.d.	1.27 ± 0.02	n.d.	n.d.	4.54 ± 0.02	n.d.	42.32 ± 0.54
14	Heptanal	905	906	1.42 ± 0.01	4.31 ± 0.07	n.d.	8.19 ± 0.05	n.d.	n.d.	n.d.	n.d.
15	(2E)-Heptenal	958	956	n.d.	2.88 ± 0.04	1.99 ± 0.02	n.d.	n.d.	n.d.	2.32 ± 0.02	1.24 ± 0.02
16	Benzaldehyde	962	960	0.81 ± 0.01	n.d.	n.d.	3.52 ± 0.04	6.33 ± 0.07	n.d.	6.62 ± 0.08	n.d.
17	Heptanol	971	970	n.d.	n.d.	0.80 ± 0.02	n.d.	2.42 ± 0.02	n.d.	1.71 ± 0.03	n.d.
18	1-Octen-3-ol	978	978	n.d.	n.d.	n.d.	n.d.	6.18 ± 0.04	n.d.	6.49 ± 0.05	1.22 ± 0.02
19	Hexanoic acid	982	979	n.d.	n.d.	2.59 ± 0.05	n.d.	3.01 ± 0.02	n.d.	n.d.	10.30 ± 0.10
20	2-Octanone	991	989	2.93 ± 0.02	n.d.	2.17 ± 0.03	n.d.	n.d.	n.d.	n.d.	n.d.
21	Octanal	1008	1006	2.33 ± 0.01	2.23 ± 0.04	n.d.	2.82 ± 0.02	5.38 ± 0.02	n.d.	2.87 ± 0.03	n.d.
22	(2E,4E)-Heptadienal	1016	1013	2.71 ± 0.02	n.d.	n.d.	8.50 ± 0.05	n.d.	n.d.	n.d.	n.d.
23	Limonene	1032	1030	n.d.	2.85 ± 0.06	n.d.	2.35 ± 0.02	n.d.	n.d.	2.34 ± 0.02	n.d.
24	Octanol	1076	1076	n.d.	n.d.	0.98 ± 0.02	n.d.	n.d.	n.d.	2.07 ± 0.02	n.d.
25	2-Nonanone	1093	1090	n.d.	n.d.	1.06 ± 0.02	n.d.	5.30 ± 0.02	n.d.	5.42 ± 0.05	n.d.
26	Nonanal	1112	1107	4.80 ± 0.02	n.d.	2.73 ± 0.03	5.38 ± 0.03	6.00 ± 0.03	7.99 ± 0.13	5.09 ± 0.04	n.d.
27	Octanoic acid	1196	1192	n.d.	n.d.	15.16 ± 0.18	n.d.	1.47 ± 0.01	n.d.	n.d.	3.89 ± 0.06
28	Ethyl octanoate	1203	1202	n.d.	n.d.	7.39 ± 0.10	n.d.	3.27 ± 0.02	n.d.	n.d.	n.d.
29	Decanal	1210	1208	n.d.	2.58 ± 0.04	n.d.	n.d.	4.27 ± 0.02	n.d.	n.d.	1.04 ± 0.01
30	(2E,4E)-Nonadienal	1221	1218	7.75 ± 0.04	n.d.	n.d.	n.d.	n.d.	n.d.	8.97 ± 0.09	n.d.
31	(2E)-Decenal	1270	1265	1.11 ± 0.01	10.50 ± 0.19	n.d.	n.d.	n.d.	n.d.	n.d.	n.d.
32	(2E,4E)-Decadienal	1323	1322	10.68 ± 0.04	8.89 ± 0.14	n.d.	n.d.	n.d.	n.d.	3.18 ± 0.04	1.19 ± 0.02
33	(E)-Caryophyllene	1428	1424	n.d.	0.75 ± 0.01	n.d.	n.d.	2.36 ± 0.03	n.d.	n.d.	1.03 ± 0.01

RI ^¤^ = Retention Indices measured against a mixture of C8–C18 n-alkanes on a Zebron-5ms column. RI ^†^ = Published Retention Indices measured on a 5% diphenyl- stationary phase (source: FFNSC 2 library). n.d. = not determined.

## Data Availability

Data is contained within the article (or Supplementary Materials).

## Supplementary Materials

The following are available online at https://www.mdpi.com/article/10.3390/foods11101386/s1, Table S1: Uncertainty values for all the volatiles determined in the flavor fingerprints.
