# Supplementary material for: Exploring Lignans, a Class of Health Promoting Compounds, in a Variety of Edible Oils from Brazil
Source: Foods. 2022 May 11;11(10):1386. doi: 10.3390/foods11101386 (PMC9141677; doi:10.3390/foods11101386)
Supplement: Supplementary file 1 [file foods-11-01386-s001.zip › foods-1693456-supplementary-done.pdf]

foods

MDPI

Table S1. Uncertainty values for all the volatiles determined in the flavor fingerprints.

nr.	compound	RI[¤]	RI[†]	Precision and Accuracy	Canola (mg/Kg)	Avocado (mg/Kg)	Coconut (mg/Kg)	Palm (mg/Kg)	Grapeseed (mg/Kg)	Macadamia (mg/Kg)	Brazil nut (mg/Kg)	Pequi (mg/Kg)
1	Butanal	*610*	*607*	$\bar{x}$ ± SD	2.02±0.01	2.76±0.04	n.d.	5.53±0.04	n.d.	n.d.	n.d.	n.d.
				SE	0.004	0.02		0.02				
				Asimmetry	1.73	1.62		0.64				
				Expanded Uncertainty (confidence level= 95%)	0.02	0.10		0.09				
2	Butanol	*620*	*617*	$\bar{x}$ ± SD	n.d.	n.d.	1.23±0.02	n.d.	n.d.	n.d.	5.21±0.04	n.d.
				SE			0.011				0.01	
				Asimmetry			1.32				1.72	
				Expanded Uncertainty (confidence level= 95%)			0.04				0..09	
3	2-Methylbutanal	*663*	*662*	$\bar{x}$ ± SD	3.42±0.03	n.d.	n.d.	4.38±0.02	n.d.	n.d.	n.d.	n.d.
				SE	0.01			0.010				
				Asimmetry	1.69			-1.73				
				Expanded Uncertainty (confidence level= 95%)	0.08			0.04				
4	2,3-Pentanedione	*699*	*695*	$\bar{x}$ ± SD	6.12±0.02	n.d.	n.d.	n.d.	n.d.	12.36±0.03	n.d.	n.d.
				SE	0.012					0.01		
				Asimmetry	1.09					1.03		
				Expanded Uncertainty (confidence level= 95%)	0.05					0.06		
5	Pentanal	*701*	*696*	$\bar{x}$ ± SD	1.64±0.01	1.80±0.03	n.d.	2.33±0.04	n.d.	n.d.	n.d.	n.d.
				SE	0.03	0.015		0.02				
				Asimmetry	1.68	1.34		1.16				
				Expanded Uncertainty (confidence level= 95%)	0.015	0.06		0.09				
6	Acetoin	*721*	*716*	$\bar{x}$ ± SD	n.d.	3.65±0.06	7.78±0.14	n.d.	n.d.	n.d.	n.d.	5.84±0.06
				SE		0.03	0.08					0.07
				Asimmetry		1.43	-1.69					-1.59
				Expanded Uncertainty (confidence level= 95%)		0.15	0.35					0.13
7	Isopentyl alcohol	*728*	*729*	$\bar{x}$ ± SD	n.d.	n.d.	0.72±0.01	n.d.	1.79±0.02	n.d.	n.d.	n.d.
				SE			0.003		0.01			
				Asimmetry			1.55		1.13			
				Expanded Uncertainty (confidence level= 95%)			0.03		0.04			

**8**	Pentanol	*754*	*759*	$\bar{x}$ ± SD	n.d.	n.d.	1.12±0.03	n.d.	n.d.	n.d.	1.66±0.02	n.d.
				SE			0.01				0.01	
				Asimmetry			1.63				1.68	
				Expanded Uncertainty (confidence level= 95%)			0.06				0.04	
**9**	2,3-Butanediol	*792*	*788*	$\bar{x}$ ± SD	n.d.	2.02±0.03	5.55±0.20	n.d.	n.d.	n.d.	n.d.	n.d.
				SE			0.11					
				Asimmetry			1.52					
				Expanded Uncertainty (confidence level= 95%)			0.48					
**10**	Hexanal	*801*	*801*	$\bar{x}$ ± SD	1.91±0.01	19.28±0.32	3.33±0.04	9.35±0.06	n.d.	n.d.	12.31±0.12	n.d.
				SE	0.004	0.18	0.02	0.03			0.01	
				Asimmetry	1.64	1.36	0.90	0.24			0.98	
				Expanded Uncertainty (confidence level= 95%)	0.020	0.80	0.09	0.15			0.25	
**11**	2-Hexanol	*804*	*802*	$\bar{x}$ ± SD	n.d.	0.87±0.01	n.d.	n.d.	n.d.	n.d.	16.53±0.16	n.d.
				SE		0.008					0.01	
				Asimmetry		1.73					1.29	
				Expanded Uncertainty (confidence level= 95%)		0.03					0.32	
**12**	(2E)-Hexenal	*853*	*850*	$\bar{x}$ ± SD	0.89±0.00	n.d.	n.d.	5.63±0.04	n.d.	n.d.	n.d.	n.d.
				SE	0.002			0.02				
				Asimmetry	-0.71			1.12				
				Expanded Uncertainty (confidence level= 95%)	0.009			0.10				
**13**	2-Heptanone	*902*	*898*	$\bar{x}$ ± SD	n.d.	n.d.	1.27±0.02	n.d.	n.d.	4.54±0.02	n.d.	42.32±0.54
				SE			0.009			0.02		0.02
				Asimmetry			-0.30			-0.18		1.28
				Expanded Uncertainty (confidence level= 95%)			0.03			0.04		2.15
**14**	Heptanal	*905*	*906*	$\bar{x}$ ± SD	1.42±0.01	4.31±0.07	n.d.	8.19±0.05	n.d.	n.d.	n.d.	n.d.
				SE	0.003	0.04		0.03				
				Asimmetry	1.71	1.55		0.20				
				Expanded Uncertainty (confidence level= 95%)	0.016	0.17		0.13				
**15**	(2E)-Heptenal	*958*	*956*	$\bar{x}$ ± SD	n.d.	2.88±0.04	1.99±0.02	n.d.	n.d.	n.d.	2.32±0.02	1.24±0.02
				SE		0.02	0.01				0.02	0.01
				Asimmetry		1.36	1.23				1.73	1.49
				Expanded Uncertainty (confidence level= 95%)		0.10	0.05				0.04	0.04
**16**	Benzaldehyde	*962*	*960*	$\bar{x}$ ± SD	0.81±0.01	n.d.	n.d.	3.52±0.04	6.33±0.07	n.d.	6.62±0.08	n.d.
				SE	0.004			0.02	0.04		0.04	
				Asimmetry	0.96			0.78	-1.04		-1.09	
				Expanded Uncertainty	0.019			0.10	0.17		0.16	

				(confidence level= 95%)								
**17**	Heptanol	*971*	*970*	$\bar{x}$ **± SD**	n.d.	n.d.	0.80±0.02	n.d.	2.42±0.02	n.d.	1.71±0.03	n.d.
				SE			0.01		0.00		0.03	
				Asimmetry			1.40		-1.73		-1.56	
				Expanded Uncertainty (confidence level= 95%)			0.05		0.04		0.06	
**18**	1-Octen-3-ol	*978*	*978*	$\bar{x}$ **± SD**	n.d.	n.d.	n.d.	n.d.	6.18±0.04	n.d.	6.49±0.05	1.22±0.02
				SE			0.03		0.02		0.03	0.01
				Asimmetry			1.72		-1.39		-1.24	1.29
				Expanded Uncertainty (confidence level= 95%)			0.130		0.09		0.12	0.04
**19**	Hexanoic acid	*982*	*979*	$\bar{x}$ **± SD**	n.d.	n.d.	2.59±0.05	n.d.	3.01±0.02	n.d.	n.d.	10.30±0.10
				SE			0.03		0.01			0.03
				Asimmetry			1.72		1.12			1.78
				Expanded Uncertainty (confidence level= 95%)			0.130		0.05			
**20**	2-Octanone	*991*	*989*	$\bar{x}$ **± SD**	2.93±0.02	n.d.	2.17±0.03	n.d.	n.d.	n.d.	n.d.	n.d.
				SE	0.008		0.018					
				Asimmetry	1.50		1.68					
				Expanded Uncertainty (confidence level= 95%)	0.038		0.08					
**21**	Octanal	*1008*	*1006*	$\bar{x}$ **± SD**	2.33±0.01	2.23±0.04	n.d.	2.82±0.02	5.38±0.02	n.d.	2.87±0.03	n.d.
				SE	0.006	0.02		0.010	0.01		0.01	
				Asimmetry	0.001	1.63		0.63	0.32		0.68	
				Expanded Uncertainty (confidence level= 95%)	0.02	0.10		0.04	0.05		0.06	
**22**	(2E,4E)-Heptadienal	*1016*	*1013*	$\bar{x}$ **± SD**	2.71±0.02	n.d.	n.d.	8.50±0.05	n.d.	n.d.	n.d.	n.d.
				SE	0.02			0.03				
				Asimmetry	1.29			-0.91				
				Expanded Uncertainty (confidence level= 95%)	0.09			0.13				
**23**	Limonene	*1032*	*1030*	$\bar{x}$ **± SD**	n.d.	2.85±0.06	n.d.	2.35±0.02	n.d.	n.d.	2.34±0.02	n.d.
				SE		0.03		0.00			0.02	
				Asimmetry		1.69		-1.73			1.15	
				Expanded Uncertainty (confidence level= 95%)		0.13		0.04			0.04	
**24**	Octanol	*1076*	*1076*	$\bar{x}$ **± SD**	n.d.	n.d.	0.98±0.02	n.d.	n.d.	n.d.	2.07±0.02	n.d.
				SE			0.01				0.01	
				Asimmetry			1.62				1.73	
				Expanded Uncertainty (confidence level= 95%)			0.04				0.05	
**25**	2-Nonanone	*1093*	*1090*	$\bar{x}$ **± SD**	n.d.	n.d.	1.06±0.02	n.d.	5.30±0.02	n.d.	5.42±0.05	n.d.
				SE			0.008		0.01		0.02	
				Asimmetry			1.63		-1.36		-1.39	

			Expanded Uncertainty (confidence level= 95%)			0.03		0.06		0.12	
**26**	Nonanal	*1112 1107*	**$\bar{x}$ ± SD**	4.80±0.02	n.d.	2.73±0.03	5.38±0.03	6.00±0.03	7.99±0.13	5.09±0.04	n.d.
			SE	0.01		0.019	0.01	0.01	0.01	0.01	
			Asimmetry	1.47		-0.61	1.47	-1.67	1.24	1.78	
			Expanded Uncertainty (confidence level= 95%)	0.04		0.08	0.06	0.07	0.26	0.08	
**27**	Octanoic acid	*1196 1192*	**$\bar{x}$ ± SD**	n.d.	n.d.	15.16±0.18	n.d.	1.47±0.01	n.d.	n.d.	3.89±0.06
			SE			0.10		0.00			0.02
			Asimmetry			1.67		1.73			1.54
			Expanded Uncertainty (confidence level= 95%)			0.44		0.03			0.13
**28**	Ethyl octanoate	*1203 1202*	**$\bar{x}$ ± SD**	n.d.	n.d.	7.39±0.10	n.d.	3.27±0.02	n.d.	n.d.	n.d.
			SE			0.05		0.01			
			Asimmetry			1.28		-1.71			
			Expanded Uncertainty (confidence level= 95%)			0.23		0.04			
**29**	Decanal	*1210 1208*	**$\bar{x}$ ± SD**	n.d.	2.58±0.04	n.d.	n.d.	4.27±0.02	n.d.	n.d.	1.04±0.01
			SE		0.02			0.01			0.01
			Asimmetry		1.58			-1.18			-0,79
			Expanded Uncertainty (confidence level= 95%)		0.11			0.05			0.02
**30**	(2E,4E)-Nonadienal	*1221 1218*	**$\bar{x}$ ± SD**	7.75±0.04	n.d.	n.d.	n.d.	n.d.	n.d.	8.97±0.09	n.d.
			SE	0.02						0.02	
			Asimmetry	1.29						1.34	
			Expanded Uncertainty (confidence level= 95%)	0.09						0.17	
**31**	(2E)-Decenal	*1270 1265*	**$\bar{x}$ ± SD**	1.11±0.01	10.50±0.19	n.d.	n.d.	n.d.	n.d.	n.d.	n.d.
			SE	0.003	0.10						
			Asimmetry	-0.59	1.58						
			Expanded Uncertainty (confidence level= 95%)	0.013	0.46						
**32**	(2E,4E)-Decadienal	*1323 1322*	**$\bar{x}$ ± SD**	10.68±0.04	8.89±0.14	n.d.	n.d.	n.d.	n.d.	3.18±0.04	1.19±0.02
			SE	0.02	0.07					0.01	0.02
			Asimmetry	-0.70	1.32					-0.75	-0.68
			Expanded Uncertainty (confidence level= 95%)	0.110	0.33					0.08	0.04
**33**	(E)-Caryophyllene	*1428 1424*	**$\bar{x}$ ± SD**	n.d.	0.75±0.01	n.d.	n.d.	2.36±0.03	n.d.	n.d.	1.03±0.01
			SE		0.004			0.01			0.01
			Asimmetry		1.67			0.81			0.88
			Expanded Uncertainty (confidence level= 95%)		0.018			0.08			0.02
